# Multiscale modeling in the clinic: diseases of the brain and nervous system

**DOI:** 10.1007/s40708-017-0067-5

**Published:** 2017-05-09

**Authors:** William W. Lytton, Jeff Arle, Georgiy Bobashev, Songbai Ji, Tara L. Klassen, Vasilis Z. Marmarelis, James Schwaber, Mohamed A. Sherif, Terence D. Sanger

**Affiliations:** 1 Department of Physiology and Pharmacology and Neurology, SUNY Downstate, Kings County Hospital, Brooklyn, NY 11203 USA; 2 Harvard U, Cambridge, MA USA; 3 RTI International, Durham, NC USA; 4 Thayer School of Engineering, Department of Surgery and of Orthopaedic Surgery, Geisel School of Medicine, Dartmouth College, Hanover, NH 3755 USA; 5 U British Columbia, Vancouver, BC Canada; 6 USC, Los Angeles, CA USA; 7 Jefferson U, Philadelphia, PA USA; 8 Yale U, New Haven, CT USA; 9 VA Connecticut Healthcare System, West Haven, CT USA; 10 Ain Shams U Institute of Psychiatry, Cairo, Egypt

**Keywords:** Multiscale computer modeling, Simulation, Schizophrenia, Drug addiction, Neurorehabilitation, Neurostimulation, Stroke, Epilepsy, Traumatic brain injury

## Abstract

Computational neuroscience is a field that traces its origins to the efforts of Hodgkin and Huxley, who pioneered quantitative analysis of electrical activity in the nervous system. While also continuing as an independent field, computational neuroscience has combined with computational systems biology, and neural multiscale modeling arose as one offshoot. This consolidation has added electrical, graphical, dynamical system, learning theory, artificial intelligence and neural network viewpoints with the microscale of cellular biology (neuronal and glial), mesoscales of vascular, immunological and neuronal networks, on up to macroscales of cognition and behavior. The complexity of linkages that produces pathophysiology in neurological, neurosurgical and psychiatric disease will require multiscale modeling to provide understanding that exceeds what is possible with statistical analysis or highly simplified models: how to bring together pharmacotherapeutics with neurostimulation, how to personalize therapies, how to combine novel therapies with neurorehabilitation, how to interlace periodic diagnostic updates with frequent reevaluation of therapy, how to understand a physical disease that manifests as a disease of the mind. Multiscale modeling will also help to extend the usefulness of animal models of human diseases in neuroscience, where the disconnects between clinical and animal phenomenology are particularly pronounced. Here we cover areas of particular interest for clinical application of these new modeling neurotechnologies, including epilepsy, traumatic brain injury, ischemic disease, neurorehabilitation, drug addiction, schizophrenia and neurostimulation.

## Introduction

**Fig. 1:**
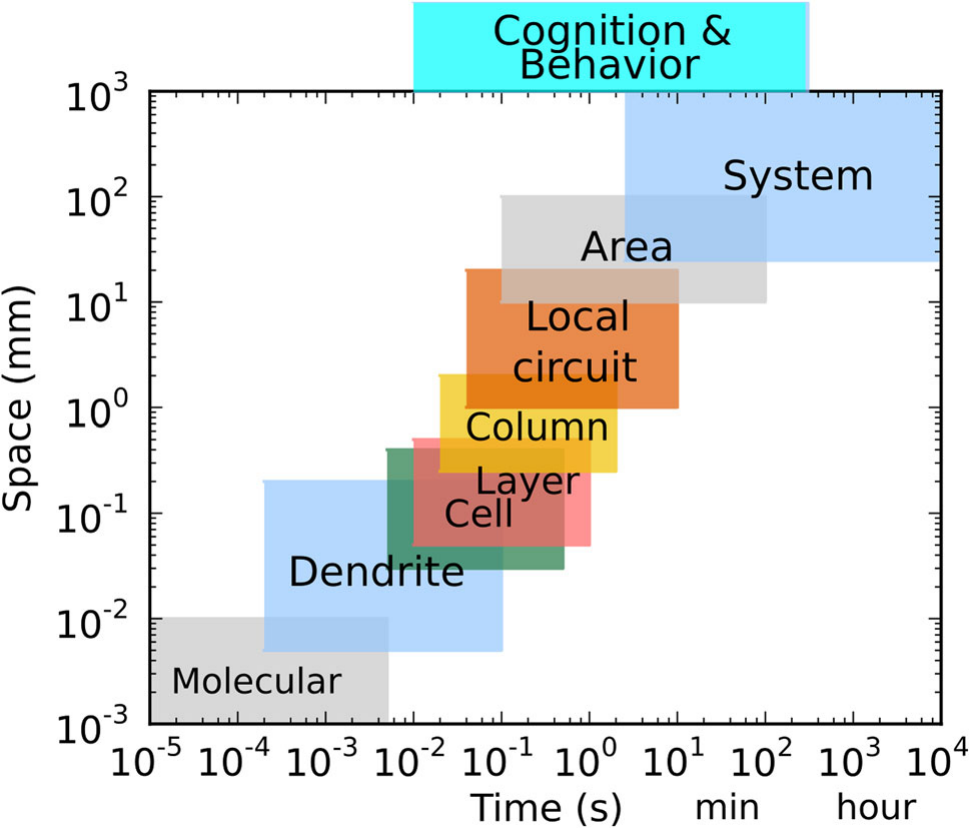
Temporal and spatial scales of organization in the nervous system. The proper spatial scale of “Cognition and Behavior” depends on how it is being viewed and modeled. Scale overlap can be seen by noting that dendrite, cell and column share scale in both time and space, reflecting the fact that the same neural signals are being processed at these different levels

The brain is the most complex organ in the body. Molecular and cellular-level processes combine into populations of neurons connected through brain systems and subsystems to generate behaviors that range from simple movements to social interactions. Scales range from the molecular scale of ion channels and pharmacological agents to scales of interconnectivity across brain areas and beyond with interactions that sometimes skip across scales (Fig. 1). The multiplicity and interconnectivity of these scales requires a multiscale modeling approach to provide understanding of brain function and brain disorders.

Multiscale modeling (MSM) of diseases of the nervous system is particularly challenging due to a number of factors. First, brain MSM differs from MSM of other organ systems due both to scale extension and to scale overlap (Fig. 1). The highest scales of interest for the brain, cognition and behavior are of great interest but are particularly resistant to study. These can be measured in temporal scale through reaction times. Cognitive processes can also be investigated using several indirect measures, particularly those of information theory. The spatial scales of the brain basis of behavior are less clear, though one can attach aspects of behaviors to particular brain areas, as with the dissection of the language faculty into Wernicke’s and Broca’s areas. For clinical application, it is valuable to extend these models still further (“above the skin”) in order to connect to models that are developed to look at social interactions (e.g., in addiction studies) and epidemiology.

A second unusual modeling challenge comes from the overlap across scales in the brain. In some modeling areas, one can perform a series of model encapsulations, providing a reduced model at each scale that can then be plugged into a new model at the higher scale. This approach produces a multiscale modeling via stepwise embedding. The use of this encapsulating/embedding approach in brain modeling is limited by the overlap across scales. For example, an important scale overlap occurs at cell to network scales: A pyramidal cell apical dendrite (a subcellular structure) spans 100s of microns, crossing multiple laminae of cortical network. This dendrite will integrate signals at both spatial and temporal scales similar to those being handled by multiple neurons in multiple interconnected non-encapsulatable networks. In practice, brain modelers often do encapsulate, for example making the practical decision to treat the individual neuron in the network as a point neuron. However, this, and other, encapsulating approximations, like all approximations, represent a trade-off of detailed representation versus conceptual clarity.

Thirdly, multiscale modeling for brain and nervous system disease has developed out of an older field, computational neuroscience, that is only now adding computational systems biology techniques to its historical focus on cellular electrophysiology and abstract networks. Adding the molecular scale, newer models now identify and investigate chemical signaling cascades, many synaptically triggered via metabotropic receptors, to the traditional assessment of electrical signaling via ionotropic receptors. One particular focus has been on the role of calcium, a second messenger signal that can be released from endoplasmic reticulum stores by calcium-induced calcium release (CICR), as well as from extracellular stores [1, 2]. Inclusion of chemical modeling is particularly valuable for improving our understanding of pharmacotherapeutics.

Fourth, there are a large number of disorders and diseases of the nervous system, many of which are not purely brain diseases but instead involve interactions with other systems that are the primary source of pathology. Importantly, a stroke damages the nervous system due to vascular pathology. However, as described below, stroke modeling has either focused on the brain and excluded consideration of blood vessels and the heart or looked at the vasculature but not included neural tissue in any detail. Similarly, multiple sclerosis is a disease involving interaction of the immune system and the brain.

Finally, in addition to involving multiple systems and multiple scales, understanding brain disease requires multialgorithmic and multiphysics approaches that may be needed at one particular scale or for one particular problem. Multialgorithmically, techniques from graph theory are used at scales from microcircuit up to connectomics among areas of the brain. Techniques from information theory are used for analysis of spike trains at relatively fast temporal scales, but are less useful for slower oscillations and other types of brain signals. With respect to multiphysics, finite element (FE) modeling is utilized in two very different contexts pertaining to different clinical scenarios. FE of electrical signals in bulk brain tissue is used for understanding electrostimulation therapy. FE of spread of pressure waves through the brain is used in study of traumatic brain injury.

Due to the broad scope of this field and the preliminary nature of much of the research, we cover only a few selected disorders and diseases. For each disease, we highlight specific research questions which could be answered using multiscale modeling. We then focus on the attempts made at bridging the scales, as well as the many challenges which must be met to provide clinically useful models. Our target audience is clinicians in psychiatry, neurology, neurosurgery and physiatry (rehabilitation medicine), as well as investigators involved in neural modeling.

## Epilepsy

Many years of progress in basic science and modeling of epilepsy have evaluated this disease as an “electrical storm” in the brain whose causes involve enhanced excitability of cells and synapses [3, 4]. However, additional biological scales also contribute to etiology and pathogenesis of epilepsy, ranging from individual ion channels (channelopathies), to individual cells, to networks. Mutations in the genes encoding ion channels, including missense mutations and premature stop codons, alter the dynamics of the ion channels [5, 6]. Such changes can result in either inherited or sporadic epilepsy. In the sporadic epilepsy case, the gene mutation or allelic predisposition is only one of many factors at multiple scales which produce the acquired disorder. Genomics influences electrical and chemical dynamics producing local and global network effects with alterations in behavior.

Mutations affecting ion channels can be used to investigate the function of the different domains of the ion channel protein (for example, formation of channel pore or voltage sensors). Using homology modeling, the 3D structure of the protein is predicted based on homology of amino acid sequences of proteins with known 3D structures. This is then combined with calculations of free-energy minimization to optimize packing of the polypeptide sequence [7]. These models can be used to test the influence of various mutations on the dynamics of the ion channels. Effects could then be reflected in the mathematical functions representing the behavior of the ion channels. Inserting such channels into cellular and network models results in changes in the excitability of the cells (e.g., the duration of action potential, and the frequency of spiking) [8, 9].

On the other end of spatial scale are whole brain regions. Modeling large regions has become increasingly popular in light of the use of brain stimulation as a treatment modality. Older, more detailed models have led to some insights in seizure attractor states and underlying propagation dynamics, but new devices such as vagus nerve stimulators (VNS) and closed-loop brain stimulation systems make it important to understand underlying physiology as well as gross dynamics [10]. Simulation of brain stimulation for epilepsy has substantial overlap with work being done for other disorders (see Sect. 8 below). VNS study overlaps with stimulation of peripheral nerves [11]. However, in the case of VNS the stimulation is done in order to produce secondary effects in cortex—these intracerebral changes remain poorly understood.

## Traumatic brain injury

Modeling the effects of trauma on the brain requires both top-down and bottom-up approaches. The top-down approach describes the distribution of force and energy on various brain regions, while the bottom-up approach describes effects of the trauma at the micro- and nanoscales. Multiscale models bridge the gap between various types of injury and the damage seen through neuroimaging. It has been shown that model-estimated brain regional responses are more effective in injury prediction than kinematics-based injury metrics [12–14].

Biomechanical modeling of traumatic brain injury (TBI) uses finite element (FE) models of the human head to understand how energy from external head impact is converted into the regional mechanical responses that cause focal brain injury [15–17]. Finite element modeling is used in both TBI research and in assessing the spread of electrical signaling in neurostimulation research (see Sect. 8 below). Although the numerical techniques are similar, the physical parameters involved in these two domains are vastly different—mechanical versus electrical parameters. However, structural neuroimaging can be used to suggest pathways both for mechanical stress and for electrical conductivity.

Numerous head mechanical models have been developed in the past several decades that vary significantly in model features and parameters [17, 18] . Along with the evolution of model development, significant efforts have been made to better characterize brain material properties to improve these models by considering inter-regional heterogeneity across gray and white matter and white matter material anisotropy [19–21]. However, different head models produce significantly different results in terms of strain and stress, even under identical impact conditions [18]. This model dependency must be considered when interpreting and comparing results from different research groups.

Damage to white matter pathways is particularly important in determining clinical symptoms of TBI due to diffuse axonal injury. Standard isotropic response analyses (maximum principal strain) do not account for white matter structural anisotropy and are not able to characterize shearing and elongation. Recent studies have begun to incorporate axonal strain directly, improving injury prediction [12, 22–27]. However, greater resolution is needed to estimate fiber strains at subvoxel resolution and to assess fiber strains along their entire length [28]. This is important, as assessing fiber strains along the entire length of fibers enables assessing the injury risks to white matter neural pathways or tracts, which is not possible with element/voxel-based studies. Incorporating whole-brain tractography into multiscale models permits graph theoretic prediction at the level of the structural connectome [29]. By incorporating axonal cell death models [30], damage to functional brain networks can be computed. This potentially offers a physics-based insight into the biomechanical and neurophysiological mechanisms of TBI symptomatology.

The top-down modeling described above does not incorporate structures of the brain at cellular and tissue scales. Bottom-up approaches have been used to simulate tissue responses at the micro- and nanoscale and to incorporate models of axonal cell death from in vitro studies [27, 30–32]. Local tissue- and cell-scale damage due to TBI is similar to that of ischemia, with involvement of apoptosis and necrosis (see next section). Top down and bottom up could be combined by using a whole-brain model of impact at the macroscale with microscale simulation targeted at the brain regions maximally affected, using the boundary conditions derived from the macromodel [12], a model encapsulation technique.

Most head models in TBI impact simulations are based on a 50th percentile adult head [25]. These are effective for population-based studies but do not incorporate individual anatomy and individualized axonal structural anisotropy. Multiscale, personalized head impact simulations, incorporating individualized imaging, will become important for precision or personalized brain injury treatment and prognostication.

## Ischemia in stroke and neurodegenerative disease

A stroke is a neurological event of sudden onset due to primary problems in the vasculature. Hemorrhagic stroke, bleeding from failure of a blood vessel wall, makes up about 20% percent stroke cases. The other 80% of strokes are ischemic: tissue death due to vessel blockage, failure of blood flow and lack of metabolites. Multiscale modeling of strokes might begin with modeling of blood, blood vessels and the heart, along with the brain. Such extensive multiphysics modeling has been limited to highly idealized models. Instead, most computer modeling of stroke has assessed cellular and brain tissue effects of ischemia.

The brain has little energy reserve, so cannot tolerate loss of blood flow for long. At cellular and subcellular scales, a key focus of modeling is to determine the relative rates of signals and processes that determine whether a cell will undergo apoptosis (programmed cell death) or necrosis (rapid uncontrolled cell death). In general, cells subject to more gradual ischemia will undergo apoptosis. Apoptosis is a slower process than necrosis and does not involve release of direct cellular toxins into the intracellular space. The process of triggering apoptosis involves a long cascade involving molecules known as caspases. Modeling the caspase cascade would suggest molecular locations where one might alter the necrosis/apoptosis balance and reducing ischemic damage [33]. Another set of molecular factors with pathological implications are reactive oxygen species (ROS) [34]. The intracellular spread of these highly reactive free radicals is modeled to determine the degree of local damage that is then reflected as overall cell damage through a process referred to as oxidative stress or cell stress [35].

One goal of neural modeling for stroke is to develop neuroprotective therapies—strategies to reduce the damage immediately after a stroke occurs. For example, the role of oxygen in creating ROS helps explain why simply restoring oxygen to tissue is not generally protective. Nonetheless, it might be the case that oxygen combined with other agents could create a drug cocktail that would have neuroprotective properties. Modeling could also assess various agents that might reduce the rate of apoptosis to preserve brain tissue or alternatively enhance apoptosis to prevent the greater damage associated with necrosis.

At the next scale up, modeling places these cellular effects into the context of the surrounding tissue. Whereas most brain tissue modeling is done with reference to neuronal networks, where neurons are connected synaptically, ischemic modeling considers brain tissue in bulk with effects that are local and not synaptic. When a stroke occurs, the central area of severe ischemia is referred to as the ischemic core, and the surrounding area as the penumbra. The penumbra is of particular interest because cells there may be salvageable through timely intervention [36, 37]. Here again, modeling can make predictions as to whether necrosis or apoptosis will predominate at a particular location. Necrotic cells will release toxic contents which will spread into surrounding tissue and accelerate damage [38, 39]. This suggests how factors that influence diffusion by, for example, reducing edema could be protective.

Sudden, severe ischemia causes stroke. By contrast, prolonged low-grade metabolic insufficiency contributes to neurodegenerative disease, including Alzheimer’s disease. Hemodynamic modeling has suggested impaired vasomotor reactivity to $\hbox {CO}_2$ in early-stage Alzheimer’s patients when compared with age-matched controls [40]. These input–output hemodynamic models were estimated from beat-to-beat data of arterial blood pressure, end-tidal $\hbox {CO}_2$ and cerebral blood flow velocity measured at the middle cerebral arteries via transcranial Doppler. Pressure and $\hbox {CO}_2$ time-series data are viewed as the inputs to the model and the flow velocity data as the output. These data-based models allow the computation of indices quantifying the dynamic processes of cerebral autoregulation and $\hbox {CO}_2$ vasomotor reactivity in the individual subject. Key structural components of these models are termed “Principal Dynamic Modes” that suggest how impaired vasomotor reactivity can be related to dysregulation of potassium channels in the astrocytic membrane under conditions of elevated calcium in the astrocytic endfeet [41–43]. Subsequent modeling studies of a larger cohort of patients diagnosed with amnestic mild cognitive impairment (often a forerunner of Alzheimer’s disease) have in fact confirmed the presence of impaired $\hbox {CO}_2$ vasomotor reactivity in these patients [44].

Alzheimer’s disease not only lacks effective treatment, but also lacks reliable diagnosis. Early detection of ischemic aspects of the disease may improve diagnostic reliability and permit early treatment to reduce subsequent ischemic degeneration. As indicated above, data-based input–output hemodynamic modeling has suggested that neurodegenerative disease may be associated with impairment of vasomotor reactivity in the cerebral vasculature, likely due to dysfunction of the neurovascular unit caused by elevated calcium in the astrocytic endfeet causing dysregulation of calcium-dependent potassium channels in astrocytic membrane [45, 46]. The neurovascular unit is considered the building block of the blood brain barrier. It consists of the blood capillary endothelium, surrounding pericytes (contractile cells surrounding capillary endothelium), astrocytic endfeet, neurons and extracellular matrix. The neurovascular unit is thought to be responsible for detecting the metabolic demands of neurons and responding accordingly by vasoconstriction or vasodilatation [47]. Further study of this hypothesis about the role of the dysfunction of the neurovascular unit in neurodegenerative diseases will utilize physics-based multiscale models of the neurovascular unit to elucidate the physiological mechanisms of its responsiveness to reduced $\hbox {O}_2$ and increased $\hbox {CO}_2$ [48].

## Neurorehabilitation

Neurorehabilitation can be conceptualized as a set of techniques to facilitate the brain’s natural mechanisms for recovery from injury. The mechanisms of recovery are often limited due to the interaction between neural recovery mechanisms and the tasks the patient is performing. For example, in an unassisted recovery, the patient may restrict their environment and tasks to those that can be readily performed despite the neural deficits. But this restricted set of tasks may not challenge the brain and can be suboptimal for promoting recovery. Rehabilitation defines and encourages practice of an expanded set of tasks in order to promote more effective recovery.

In practice, neurorehabilitation becomes the focus of treatment after the acute period of injury has passed. Ideally, neurorehabilitation considerations would also inform acute treatment of stroke and traumatic brain injury. There is a need to both protect the remaining neurons from injury, as discussed above in the prior section on Ischemia, as well as to prevent early unwanted plasticity from occurring during the acute period of brain damage.

The restricted set of tasks which patients continue to make is an example of failure of motor learning, in which continued task practice does not lead to improvement. Multiscale computational modeling of motor learning based on plasticity mechanisms within populations of cells has shown two conditions where motor learning fails: (1) lack of the sensory information (or attention to the sensory information) needed for error correction; (2) large performance errors so that incorrect movements are being practiced [49]. In both cases, learning does not occur even in the presence of normal plasticity mechanisms, because either errors cannot be detected and corrected, or practice does not provide useful examples of the correct behavior. Neurorehabilitation can address both types of error at the behavioral level. For sensory errors, this can be done by focusing attention on the most important aspects of task performance. For motor difficulties, one can guide practice in an assisted environment, or practice simpler subtasks.

Brain injury is mediated by death or dysfunction at the cellular level. Recovery is thought to be mainly mediated by synaptic plasticity mechanisms. Rehabilitation therefore seeks to provide tasks to enhance plasticity that will improve function [50], using the ability of brain regions to remap and reallocate resources in response to sensory data and motor behavior [51, 52]. In addition to this role for adaptation of brain to task, there is also a role for plasticity to produce learning of strategies to perform tasks in new ways [53].

One goal of rehabilitation where multiscale modeling can help is to guide reallocation of remaining neural resources to reflect the long-term goals of the patient. This is a multiscale problem because the behavioral goals occur at the scale of body and limb movement, motor function and real-world tasks, whereas the neural remapping happens at the level of populations of neurons that are responsible for the internal representation and computation of movement. The relationship between allocation of neural resources and large-scale behavioral performance is a fundamental multiscale problem that can benefit strongly from theories that link individual and group neuron behavior to normal and abnormal body movement and skill performance. Remapping is not the only plasticity mechanism. Another, equally important, element of rehabilitation is learning new behavioral techniques to accomplish important tasks. Here, it is important to realize that fundamental synaptic plasticity mechanisms are responsible for adaptation and learning [53].

Improved understanding of how to harness plasticity for rehabilitation requires models that range from the subcellular scale of synaptic plasticity to the behavioral scale of interaction with the environment using models of adaptive and optimal control theory. Such models may segment elements of behavior into classical computational elements of control, such as optimal control, adaptive control, internal system models, Bayesian sensory observers and feedback control. This segmentation then allows a potential link to different brain regions, so that, for instance, the optimization and selection of movement may occur within one brain region (perhaps the basal ganglia) while adaptation, feedback and internal models might occur elsewhere (perhaps in the cerebellum). Once the neural systems scale has been identified, the particular behavior of groups of neurons can be measured, and predictions can be made using mathematical models of the neural effects, or by direct simulation of populations of neurons, interconnected simulated neural systems or injured neural systems. Such models can be extended even further to the microscopic scale by considering the effect on population behavior of abnormalities in membrane depolarization, perhaps due to genetic defects in ion channels or the effect of toxins on channel behavior. Thus, multiscale modeling proceeds both top-down (using theories of motor control to describe and predict high-level behavior) and bottom-up (using theories of neural computation to predict the effect of neural and neural population activity on high-level behavior). Predictions can be made across scales, so that the neural response to behavioral interventions can be predicted, and the behavioral response to neural injury can be predicted. Most important for neurorehabilitation, the neural and behavioral response to rehabilitation can be predicted, and the combined effect of therapy and medication can be predicted and tested.

Both experimental and theoretical components of the multiscale model must address the effect of the choice of sensory-motor environment and task on motor behavior and plasticity [54]. The goal is an understanding that includes both the principles of science (the ability to predict specific effects of well-controlled interventions) and engineering (the ability to build a model whose behavior emulates human rehabilitation and whose structure reflects the known neuroanatomy and neurophysiology). Iteration between multiscale modeling and experimental testing will permit the development of new therapies based on a fundamental understanding of the computational mechanisms responsible for recovery from brain injury.

## Drug addiction

Addiction is a complex psychological and neurophysiological manifestation, defined in terms of drug-using behaviors. Because of the importance of behavior in defining the syndrome, and because the syndrome depends on the availability and accessibility of the drug of abuse, which in turn depends on social interactions, it is useful to extend the concept of multiscale upwards to the levels of these social interactions [55]. Underlying mechanisms drive an individual to uncontrolled use and create feelings of craving as well as a physiological state of withdrawal. These mechanisms can be defined spatially at the level of genome to neural circuitry and temporally at multiple scales ranging from milliseconds to years, influencing each other through systems of feedback loops [56]. For example, genetics will determine the functioning of certain receptors in the brain, their response and adaptation to repeated drug intake. This adaptation in turn can gradually change cognitive pathways and lead to the intrinsic demand for more drugs, which can translate into drug-seeking behavior involving other individuals. Success in drug-seeking behavior results in drug use and reinforces across these multiscale cycles.

Addiction is addressed through several research disciplines: neurobiology, genetics, behavioral economics, epidemiology and public policy. Although mathematical modeling is applied within each of these areas, not enough modeling has attempted to cross these boundaries. Development of new strategies for treatment and prevention will require that we connect these scales, for example from rodent experiment up to epidemiology [57–59]. Approaches to plasticity to treat addiction are related to similar approaches in neurorehabilitation, in that they also attempt to take damaged internal wiring and modify it through alterations of the interactions of these circuits with the external environment.

Initial multiscale models provide a framework to describe components, processes and feedback loops spanning below the skin and above the skin [55, 60–63]. As with most of neurobiological work, animal models pave the way to theory development [57–59]. How well these theories translate to human brain is not always clear. One way is to apply rodent-based theory to a human model. Such models consider a dynamic where the neurobiology serves as a driver for behaviors, while the environment provides inputs that shape the behaviors [64]. In these models, neurobiology is much simplified because the main focus is on individual’s long-term behavior. Nevertheless, it is critical because it emphasizes the biological nature of addictive behaviors. Recent modeling proposed how drug-induced alterations of the addict’s internal physiological state may lead to a transition from drug use to addiction [65]. As more is known about individual scales—single neuron, circuitry, behavior—more challenges arise as to how to connect the pieces, because of the increasing multiplicity of pathways. These complex models run the risk of being intractable at first, but can then be simplified piecewise to derive novel hypotheses about system relationships.

## Schizophrenia

Schizophrenia plays out over the course of a lifetime. The underlying etiology is a combination of genetic/proteomic predisposition with viral or other insults during early development. This combination triggers anomalies in brain development which are only fully expressed much later, generally during the period of late adolescence. The sudden onset of psychosis at this time is known as a psychotic break, a sudden and dramatic alteration in thought patterns and behavior. The psychotic break is typically precipitated by a stressful event in the life of a predisposed individual. In many cases, this is the first episode with recurrence of further episodes of psychosis.

Using the multiscale modeling perspective, Lisman and colleagues considered that the psychotic break might reflect a bistable system with a state switch triggered by the stressful event [66]. They described this switch as being manifested in dynamic changes that would be influenced by a number of local measures, including imbalance of excitation mediated by glutamate receptors and inhibition mediated by GABA receptors (an inhibitory receptor type), abnormal gamma frequency (25–100 Hz) oscillations and hyperactive ventral tegmental area (VTA)—thalamic-hippocampal loop. One of their computer models included the CA1 region of the hippocampus, the thalamus and the VTA. CA1 was modeled as firing of principal cells which are stimulated by the bursting of thalamic neurons and inhibited by local inhibition. Bursting of thalamic cells was driven by membrane hyperpolarization, which in turn was calculated based on NMDAR (NMDA type of glutamatergic receptor) blockage and level of dopaminergic activity in the VTA. Dopaminergic activity within the VTA was dependent on input from CA1 region as well as stress. In the model, baseline reduced NMDAR activation (the predisposition) resulted in hyperpolarization of the thalamic cells, paradoxically increasing their bursting (post-anodal exaltation). In the presence of stress, activity increased in VTA and thalamus, providing positive feedback with hyperactivity. The hyperactivity persisted after the stress had been removed—a jump to an alternative attractor in this bistable system [67]. The system can subsequently make further jumps back and forth. Exposed to the same stress, a person without the NMDAR blockage predisposition would not show the switch to the alternate attractor and would maintain normal dynamics.

Another related modeling approach has focused on the role of similar NMDAR effects by looking at pharmacological NMDAR blockers that produce cognitive abnormalities similar to psychosis in normal people (some of these psychotomimetic drugs, such as ketamine and phencyclidine, are also drugs of abuse). A recent viewpoint posits abnormal cognitive coordination to be the primary underlying dysfunction in psychosis and in schizophrenia [68]. Abnormal cognitive coordination points the way to a neural substrate for thought disorder based on a proposed causal relation from neural discoordination to cognitive discoordination [69]. Evidence for anomalies in electroencephalographic (EEG) responses to cognitive tasks in schizophrenia has reinforced this notion. EEG anomalies provide the further context of brain oscillation anomalies, particularly in the gamma frequency range (30–80 Hz), as evidence of neural discoordination. Neural discoordination is based on theories of encoding through neural ensembles and neural ensemble formation through synchrony and phase locking [70]. In vivo physiology in cats suggests how synchrony between different brain regions in this gamma frequency range might allow activity in different regions to be integrated. This mechanism of coherent activation across different regions of the brain has been proposed as a possible solution to the binding problem [71], the problem of how to provide coherent object representations despite parts being widely distributed in the brain.

The presence of gamma wave anomalies after exposure to psychotomimetic drugs provides another multiscale linkage upward from the pharmacological scale. Multiscale models have assessed the effects of NMDAR blockers on cellular and network dynamics [72, 73]. In these models, the receptor changes produce oscillatory changes (network scale) in the brain with alterations in both gamma and theta (4–8 Hz) bands. The changes in gamma oscillations can be connected upwards to yet another scale by assessing the impact on information flow (functional/cognitive scale), as measured by information theory algorithms such as normalized transfer entropy. In this way, the models can help us understand the consequences of these drugs, and of schizophrenia, for altering both brain waves and thought processes.

In one of the multiscale models connecting NMDAR level to oscillations [72], a network model of the CA3 region of the hippocampus was used to examine the cellular location where NMDAR blockade would produce augmentation in gamma activity, along with reduced theta activity. The model consisted of pyramidal cells and two interneurons populations, basket cells and oriens-lacunosum moleculare (OLM) cells. The cells were Hodgkin–Huxley like conductance-based neurons, and they contained AMPAR (another type of excitatory glutamatergic receptor), NMDAR and $\hbox {GABA}_A$ receptors. The pattern of firing of the cells within the network allowed for generation of both theta and gamma oscillations. The model predicted that blocking NMDARs on OLM interneurons alone results in increased gamma and decreased theta power.

In another multiscale model, reducing NMDAR activity was associated with changes in information processing in neocortex [73]. The model contained two populations of excitatory cells (regular spiking and intrinsically bursting) and two populations of inhibitory cells (fast-spiking and low-threshold spiking), organized across the multiple layers of neocortex. These were conductance-based cellular models with three compartments—one somatic and two dendritic. NMDAR and AMPAR were located on the dendrites, while $\hbox {GABA}_A$ receptors were located on the soma. Increasing the gamma activity generated by the model permitted less information to propagate from outside into the network. This effect can be understood by noting that information in the Shannon sense is related to entropy, or degree of unpredictability, where lower predictability means higher information content. Greater stereotypy (higher predictability) in the dynamical pattern (higher gamma) meant reduced variability, leading to reduced entropy and reduced information-carrying capacity. This compares with clinical observation, where the thought pattern of patients suffering from schizophrenia shows greater stereotypical phenomena (e.g., echolalia and perseveration), associated with a reduction in global (gestalt) perceptual responsivity.

## Neurostimulation

The field of neuromodulation has been steadily growing in its breadth of treatment. A partial list of diseases treated includes chronic pain, Parkinson’s disease (PD), essential tremor, dystonia, epilepsy, depression, anxiety disorders, post-traumatic stress disorder, obsessive compulsive disease, Alzheimer’s disease, addictive substance abuse disorders and eating disorders. Neurostimulation is also used to complement the plasticity alterations produced with various physical neurorehabilitative strategies. This long list of applications suggests how the use of neurostimulation is expected to impact treatment of many of the diseases and disorders discussed above.

Over the past 15 years, advances in electrode design have allowed the development of stimulators with multiple independently driven contacts, directional contact designs, rechargeable pulse generators, wireless interfaces, enhancements in programming patterns and waveform variations, as well as simple closed-loop systems [74]. Multiscale modeling now gives us the ability to examine neural circuits on the scale of these therapies and begin to understand underlying mechanisms for the success of these currently entirely empirical treatments. Multiscale modeling allows us to examine parameter constraints, targets, waveform variations and temporal patterning, trying different ideas before moving to the animal or human platform.

Stimulation in basal ganglia is used to treat PD, depression, tremor and several other diseases. Models have extended down to the nanoscale of the electrode–tissue interface, providing understanding of the volume of tissue activated based on finite element (FE) models [75]. This has been connected to the higher scale of diffusion-tensor imaging MRI data to yield appropriate tissue electrical parameters. Knowing the volume of tissue activated in typical clinical use and combining it with connectivity revealed by DTI can allow circuitry dynamics to be modeled, since part of the imparted activation from the stimulation is now known. Further modeling has evaluated larger, neural circuitry models (column and local circuit scales) made up of thousands of biophysically detailed multicompartmental neurons (ion channel, dendrite and cell scales) with synaptic dynamics.

One model of PD consisted of basal ganglia nuclei (putamen, globus pallidus externus, subthalamic nucleus, globus pallidus internus, substantia nigra pars reticulata), substantia nigra pars compacta, thalamus and cortex [76]. PD was modeled as a reduction in activity from substantia nigra pars compacta to striatum by 80%. Increased rhythmic activity found in the thalamus, as well as in the globus pallidus internus and subthalamic nucleus, appeared similar to activity measured clinically that has been associated with the debilitating tremor of this disease. Deep brain stimulation (DBS) to the subthalamic nucleus was then simulated using a range of current amplitudes (0–3 nA) and frequencies (0–185 Hz). Certain stimulation parameters could reduce the pathological rhythmicity of these “tremor” cells. The pathological rhythms were optimally disrupted at frequency of stimulation above 135 Hz, similar to what is seen clinically.

Other scales have also been modeled. Electrode–tissue interface nanoscale models have led to better understanding of what signal or field is seen by the individual neurons. This leads to development of more efficient stimuli. Volume of tissue activated models has informed the development of multicontact steerable or directional electrodes used in DBS to reduce side effects and improve efficacy [77]. Other large-scale network models of DBS have provided understanding of the mechanisms of DBS in treating movement disorders, suggesting a role for both inhibition and excitation with changes in firing regularity in particular cell groups.

Electrodes are generally used superficially, above the dura mater, for stimulation in spinal cord and cortex. Understanding the mechanisms of spinal cord stimulation for pain hinges on understanding how the epidural field reaches the dorsal (sensory) column and its effects on cord circuitry. This multiscale problem spans ion channel subcomponent scale, up through circuitry dynamics, up to pain perception. A neural model of human spinal cord and FE models of the cord and electrodes demonstrated mechanisms of proposed stimulation paradigms prior to using them in patients [78]. This model lent support to the idea that retrograde stimulation leads to inhibition of wide-dynamic range neurons that carry pain information to the brain.

Stimulation of cortex is used to treat chronic central pain and other disorders. Currently, stimulation programming is inconsistent, particularly since benefits are not immediate but accrue over days to weeks. Multiscale modeling has been used to examine the circuit level in order to evaluate how the level of pain perception may be modulated based on alterations in network dynamics [79].

## Future directions

Scientific medicine begins to permit the development of precision and personalized medicine through opening the “black box” of disease by beginning to explain the many differences seen in patients’ responses to a particular disease or to a particular therapy. Multiscale modeling is needed because inside the black box is a complex network of interscale causal interactions. MSM thus has the potential to provide understanding of some of the problems described above. It will permit us to understand how a pharmacological intervention at the molecular level of ion channels would alter neural dynamics so as to prevent a seizure or to alter the aberrant thought process of schizophrenia. Neurostimulation is growing in importance as an empirical treatment modality with effects and consequences that remain little understood. MSM permits the linkage of these alterations in electric fields to the consequences not only on cell membranes and cell dynamics but on neural and synaptic plasticity that will produce long-term effects. Coupled with novel neurorehabilitation strategies, these techniques could then provide novel approaches for repurposing remaining brain after a patient has suffered brain damage.

Unfortunately, there remains substantial doubt about the adequacy of most models, a concern that can be traced in large part to the complexity of brain multiscale interactions described in Introduction. Most validation to date is at the level of numerical validation of the accuracy of simulation rather than experimental validation of the overall model [80]. As models increase in sophistication, they will also begin to deviate further from the basis in animal experimentation on which most model parameters are based, in order to more closely match the human condition. Although desirable to obtain these parameters directly from people, or in some cases from the individual patient, clinical measurements are severely restricted compared to what can be done in animals. Clinical experimentation is typically limited to the testing of new medications or diagnostic tests on large populations. Direct experimentation on the individual patient is also possible in very limited circumstances. For example, an epileptic patient will often be empirically treated with one drug after another, with each being titrated up to a maximum dose or till not tolerated, to find a medication that will prevent seizures in that particular patient. Ideally, multiscale modeling using individualized testing of genomic and pathologic variants will be able to reduce this kind of patient experimentation.

Two major elements of the clinical process are diagnosis and treatment. As we move toward precision and personalized medicine, these elements will be more closely allied in the individual patient. Diagnostic imaging coupled with genomic and proteomic information can be used to inform modeling in order to design the proper combination of strategies combining drugs at the chemical scale, locally or systemically, with electrical stimulation at various scales, with learning and training at the behavioral scale. As noted above, the highest scale also feeds back to the lowest: Rehabilitative training affects neuroplasticity, plasticity that will be affected by drugs and neurostimulation. Following these causal dynamical chains up and down, and back again, is the future of multiscale modeling in the brain.
